# Pediatric Multiple High-Powered Magnetic Buckyballs Ingestion—Experience From Six Tertiary Medical Centers

**DOI:** 10.3389/fpubh.2022.892756

**Published:** 2022-06-15

**Authors:** Guojian Ding, Hongzhen Liu, Peng Zhou, Qiong Niu, Wei Wang, Zhiqiang Feng, Shisong Zhang, Zhengmao Zhang, Lei Geng, Zhaoyun Bu, Tingliang Fu

**Affiliations:** ^1^Department of Pediatric Surgery, Binzhou Medical University Hospital, Binzhou, China; ^2^Department of Pediatric Surgery, Children's Hospital Affiliated to Shandong University, Jinan, China; ^3^Department of Pediatric Surgery, Zibo Maternal and Child Health Care Hospital, Zibo, China; ^4^Department of Gastroenterology, Binzhou Medical University Hospital, Binzhou, China; ^5^Department of Pediatric Surgery, Maternity and Child Health Care of Zaozhuang, Zaozhuang, China; ^6^Department of Pediatric Surgery, Taian Maternity and Child Health Hospital, Taian, China; ^7^Department of Pediatric Surgery, People's Hospital of Rizhao, Rizhao, China

**Keywords:** foreign body ingestion, high-powered magnet, Buckyballs, acute abdomen, children

## Abstract

Multiple high-powered magnetic Buckyball ingestions may lead to a high risk of severe complications. Great concerns have been raised by public health workers, and it remains challenging for clinicians to solve this troublesome problem. We report a large case series of children with Buckyball ingestion from six tertiary medical centers. The clinical data, including demographics, medical history, diagnosis tools, management options, intraoperative or endoscopic findings, and outcomes, were retrospectively analyzed. Seventy-one children aged 1–13 years ingested 2–41 Buckyballs. Among them, Buckyballs passed spontaneously on 2–10 days post-ingestion in seven cases; gastroscopic removal was performed in 14 cases; laparoscopic removal in 13 cases; laparoscopic-assisted surgical removal in 6 cases; and open surgical removal in 31 cases. Surgical indications included small bowel obstruction, perforation, peritonitis, acute abdominal pain, or along with ingestion of other metallic foreign bodies. Among those who underwent a surgical procedure, primary intestinal repair was performed in 44 cases, enterectomy with primary anastomosis in 6 cases. The postoperative hospital stay ranged from 5 to 28 days. No major complications occurred. In unwitnessed cases, a vague medical history and nonspecific symptoms usually make the diagnosis difficult. The treatment options should include the watch-and-wait approach, endoscopic, laparoscopic-assisted, or open surgical removal of Buckyballs, with primary intestinal repair or anastomosis. Preventive measures, including children's not having access to Buckyballs, are essential to protect children from this kind of unintentional injury.

## Introduction

Ingestion of foreign bodies, including coins, button batteries, bones, needles, and magnets, is one of the common unintentional injuries in children worldwide (1). Buckyball, approximately 5 mm in diameter, with high powered magnet, can steadily attract one another, even though six layers of the bowel wall apart (2, 3). Infants and toddlers usually explore objects they can touch *via* their mouth (4, 5). Ingestion of two or more Buckyballs poses a high risk of catastrophic sequelae (5). Severe alimentary tract injuries related to Buckyball ingestion, including perforation, small bowel obstruction, fistulae, peritonitis, and even life-threatening events, are increasingly reported in the past decade (6–16). Although great concerns for this preventable disease have been raised by clinicians, public health workers, and child caregivers, Buckyball ingestions in children are not uncommon in clinical practice, and this remains challenging for clinicians to solve this troublesome problem (1, 17). Herein, we present a large case series of Buckyball ingestion in pediatric patients from six tertiary medical centers, aiming to provide clinical experience in early diagnosis, rational management options, and preventive measures.

## Materials and Methods

From June 2018 to June 2021, there were 71 cases with ingestion of multiple high-powered magnetic Buckyballs at 6 tertiary medical centers. Patients' medical records, including age, gender, medical history, time since ingestion, diagnostic imaging, management options, endoscopic or intraoperative findings, and outcomes, were retrospectively analyzed. A 6-month follow-up was conducted after discharge.

## Ethical Considerations

Informed consent was obtained from the parents/legal guardian(s) of all children involved in the study.

## Results

Seventy-one children who accidentally ingested two or more Buckyballs were enrolled in this study. Of the 71 cases, there were 48 boys (69.01%). The age ranged from 1 to 13 years (the median age was 2), <3 years in 40 cases (56.31%), 3–5 years in 21 cases (29.58%), and >5 years in 10 cases (14.08%). Among females, 21/23 cases were aged 3 years or younger. The ratio was equal for both genders, with children being ≤ 3years of age (19 males, 21 females).

The number of ingestions of Buckyballs ranged from 2 to 41; the median number was 5. Seventy cases were witnessed, and one was unwitnessed. The time since ingestion of Buckyballs in the outpatient or emergency department ranged from 3 h to 1 year, including 3 h-9 days in 60 cases, ≥ 10 days in 10, >1 year in one.

**Figure 1:**
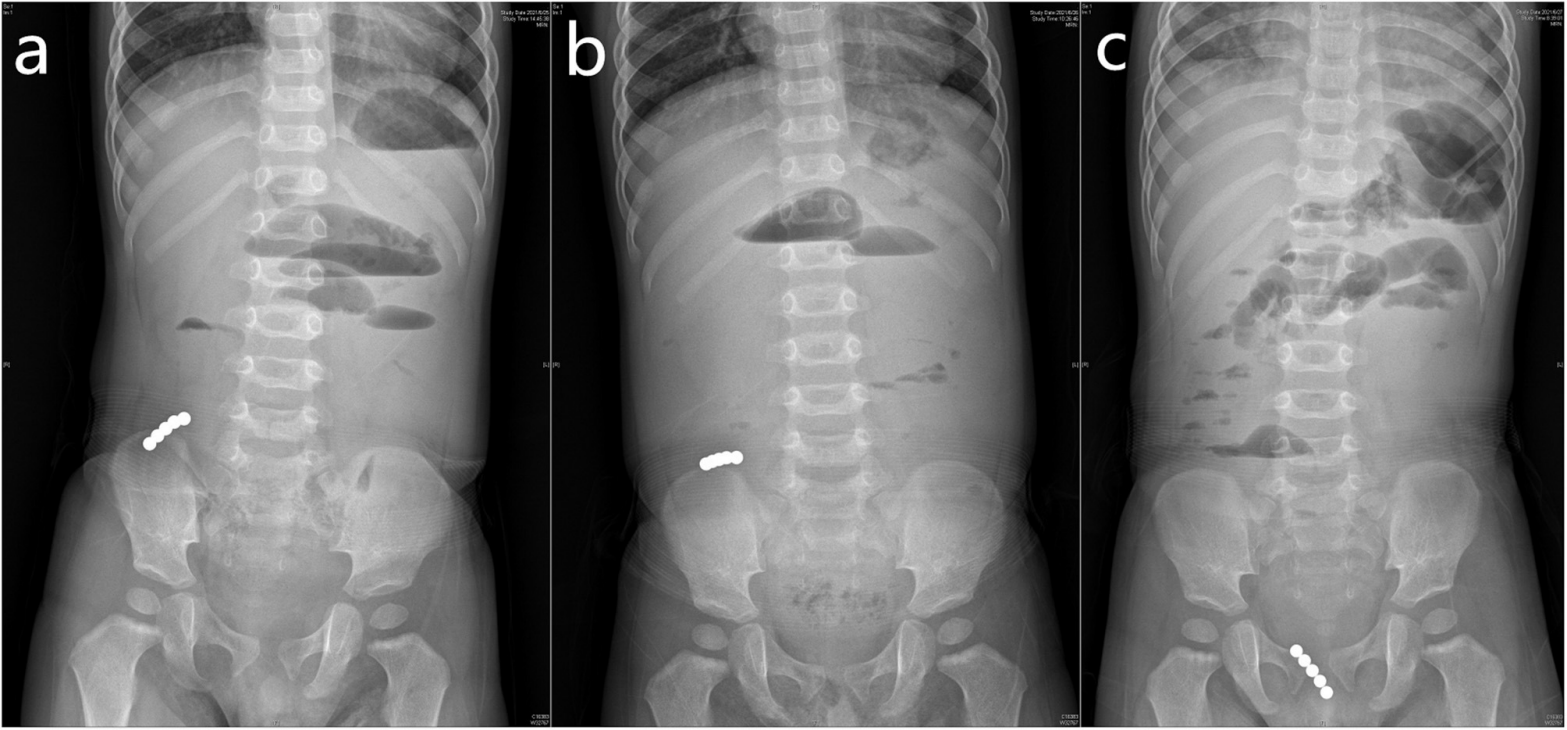
Plain abdominal and pelvic radiographic imaging revealed the progression of the Buckyballs. The Buckyballs passed spontaneously on 5 post-ingestion days and partial small bowel obstruction relieved.

All cases received plain abdominal radiography, and ingestion of 2 or more Buckyballs was confirmed. For cases planned with conservative observation, the progression of the Buckyballs was tracked by plain abdominal and pelvic radiography (Figure 1). Ultrasonographys were assessed in 45 cases. The ultrasonography revealed dilated bowel loops with bowel wall thickening, ascites, and sphere metal foreign bodies, which are located in the stomach (14), duodenum (3), small bowel (18), or colon (1), undefined localization (2). The ultrasonographic findings were consistent with the intraoperative or gastroscopic findings in 43 cases (93.33%).

**Figure 2:**
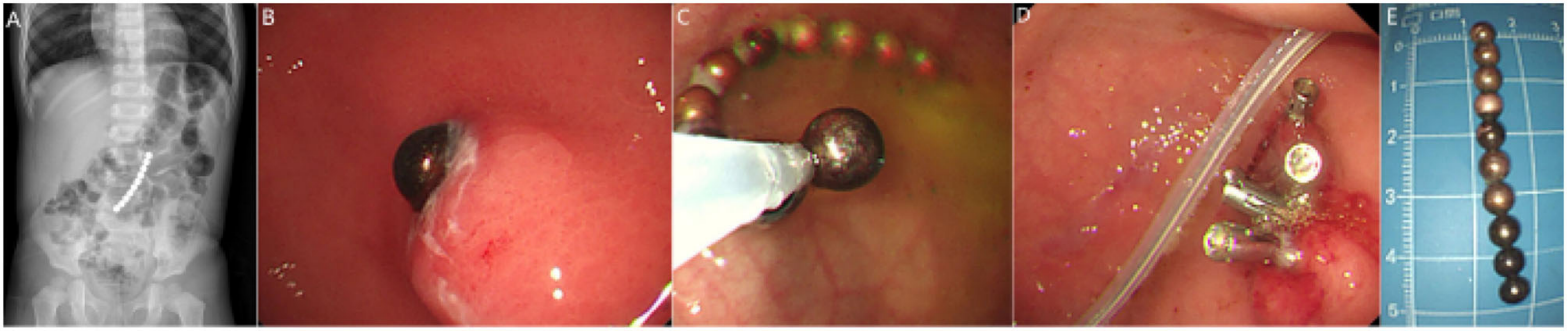
Plain abdominal radiograph showed ingestion of ten Buckyballs **(A)**. Gastroscopy revealed that only one Buckyball lodged in the stomach cavity **(B)**. Gastroscopic removal of the Buckyballs **(C,E)** and primary repair of the gastric fistula using metal closure technique **(D)** were performed, and a nasojejunal tube was inserted for early enteral nutrition **(D)**.

In all cases, ingestion of 2–4 Buckyballs passed spontaneously on the 2 to 10 post-ingestion days in seven cases (2 days in four, 3 days in two, 10 days in one). Fourteen cases received gastroscopic removal of the Buckyballs. A child with a 10-day history of ingestion of ten Buckyballs received gastroscopic removal of the Buckyballs, with primary repair of the gastric fistula *via* an endoscopic metal closure technique. A nasojejunal tube was also inserted for early enteral nutrition (Figure 2). The child recovered uneventfully without evidence of abdominal free air to suggest perforation on abdominal radiography. The remaining 50 cases underwent a surgical procedure, including laparoscopic, laparoscopic-assisted, or open surgical procedure.

Surgical indications included signs of peritonitis; bowel obstruction due to adhesion, internal hernia, or volvulus; acute abdominal pain; ring-like form, along with other metallic foreign body ingestion (button battery, screw, or iron wire, etc). The approach to the removal of Buckyballs included laparoscopic in 13 cases, laparoscopic-assisted surgical in 6 cases, and open surgical procedure in 31 cases, following primary intestinal repair in 44 cases and enterectomy, with primary intestinal anastomosis in 6 cases. The location of Buckyballs and the number and location of the perforations or fistulae were summarized in Table 1.

**Table 1:** A summary of the location of Buckyballs and perforations or fistulae.

**Buckyballs' location**	
gastro-intestine	7
gastro-intestinal-colon	3
gastro-duodenum	2
intestinal-intestine	21
intestinal-colon	8
duodenal-intestine	3
duodenum	1
duodenal-intestinal-colon	1
pelvis	1
not available	3
**Location of perforation or fistula**	
stomach	11
duodenum	4
small intestine	35
colon	14
**Number of perforation or fistula**	
2	10
3	4
4	8
5	1
6	4
9	1

The postoperative hospital stay ranged from 5 days to 28 days (median, 12 days). All cases recovered uneventfully and discharged home. No major complications occurred in a 6-month follow-up period.

## Discussion

Ingestion of a foreign body is an increasingly common clinical problem, especially in the pediatric population (1). Ingestion of two or more high-powered magnetic Buckyballs with unintentionally severe injury has been reported worldwide in the last decade (2, 5, 10, 16). It occurs most frequently in children aged 1–5 years old (19), and there is male-to-female predominance of 1.3–3.9: 1 (15, 16, 20). In the present case series, the ratio of male to female is about 2:1. However, our results showed that the ratio was equal for both genders in those aged 3 years or younger, which may provide evidence for gender differences in the prevalence in this age group.

Symptoms of ingestion of two or more Buckyballs usually emerge within 1–40 days (19, 21–23), and most cases present symptoms and signs of acute abdomen, which include onset of abdominal pain, refusal to eat, bilious vomiting, abdominal distension, dehydration, and fever.

For a prompt and precise diagnosis, investigations are needed in patients with witnessed or suspected multiple magnet ingestion (7, 12). Plain abdominal radiography may reveal the cause of small bowel obstruction of unknown origin, as our unwitnessed case. Biplane radiography, including neck, chest, abdomen, and pelvis, is essential to assess the number of Buckyballs (24) and to observe the movement of the Buckyballs and signs of potential complications, including evidence of free air or air-fluid levels in the abdomen. The ultrasonography for identification of Buckyballs is reliable and safe (25). The higher accurate rate of preoperative localization may depend on physicians' experience (as in our case series). CT scan can assess potential complications, such as a thickened bowel segment or localized pneumoperitoneum, suggesting inflammation or perforation (18, 25). Magnetic resonance imaging is strongly contraindicated due to a high risk of bowel perforation (18, 26).

The management options of ingestion of multiple Buckyball are crucial to improve patients' outcomes. Patients who present with multiple Buckyball ingestion-related complications usually require emergent surgical interventions, including laparoscopic, laparoscopic-assisted, or open surgical procedures (7, 10, 17). However, in some cases, the Buckyballs may pass through the gastrointestinal tract spontaneously under close observation (13). Watch-and-wait, close observation, and endoscopic removal may reduce the need for surgical intervention in individual patients; a well-structured management protocol needs to be elucidated (15).

As for asymptomatic patients, radiography every 12–24 h was recommended (27). Early (≤12 h) upper gastrointestinal endoscopy is recommended to retrieve Buckyballs from the stomach prior to their passage through the duodenum. Delicate manipulation is essential, while excessive force may lead to a risk of gut perforation and leak (28, 29). If the time since ingestion is >12 h and Buckyballs are suspected to have passed through the pylorus into the small bowel, a series of abdominal radiography is needed (30, 31), and surgical intervention should be considered in those who present symptoms and signs of acute abdomen. Laparoscopic surgery is an ideal approach depending on an experienced surgeon team and available facilities. The metallic tips of laparoscopic instruments may help to identify and remove the Buckyballs (8). However, in many cases, enterotomy with primary repair or bowel resection, along with primary bowel anastomosis, is needed, owing to intestinal perforation, fistulae, or bowel necrosis; a laparoscopic-assisted surgical procedure may be the choice (11). In addition, Wang et al. (2) mentioned coloscopic removal of Buckyballs. This technque may be another approach in selected cases.

**Figure 3:**
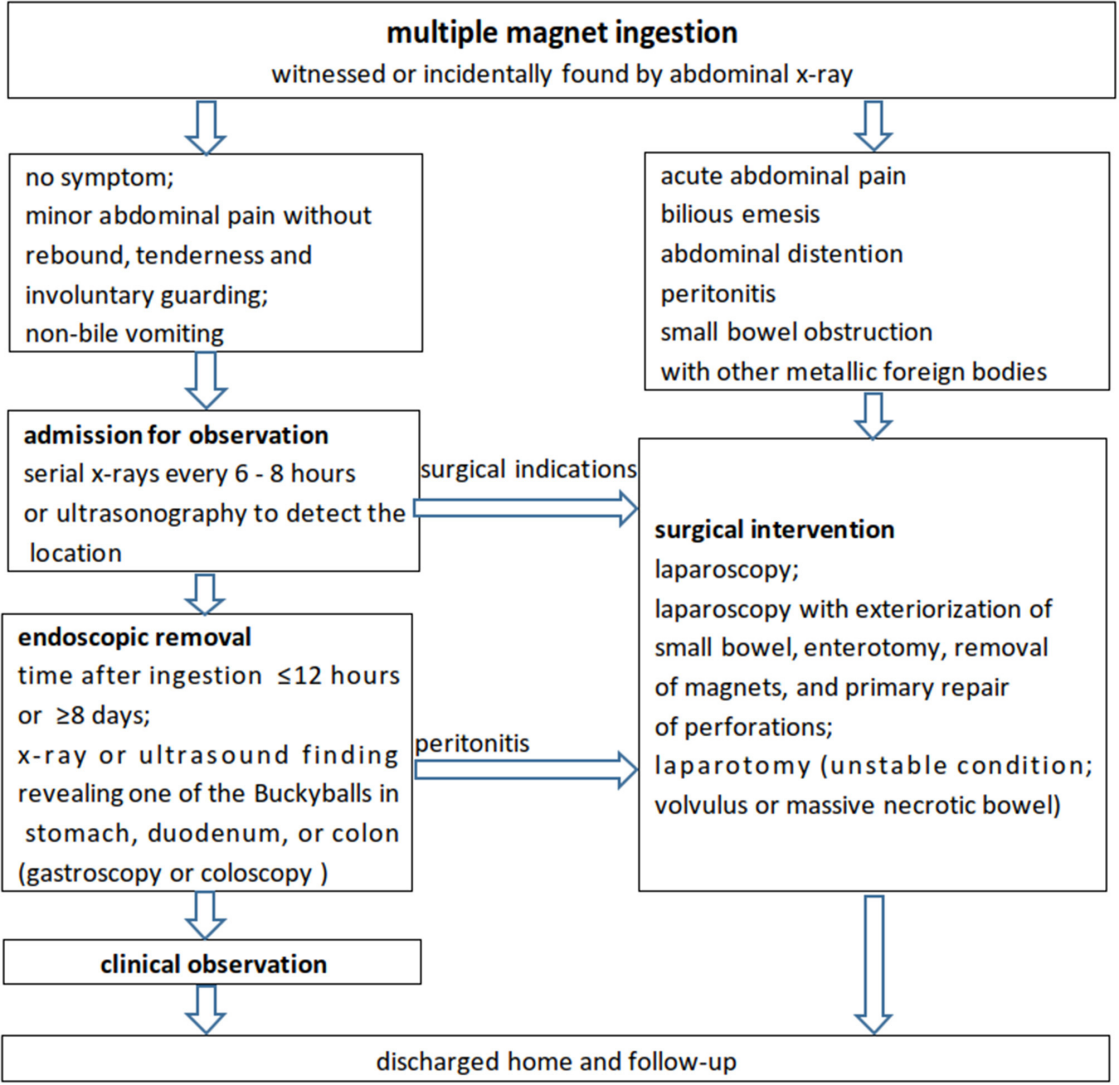
A recommended flowchart for the management of multiple high-powered magnet ingestions in the pediatric population according to the literature and our experience.

Based on the literature (1–4, 11, 14, 17, 27–29) and our multicenter experience, a flow chart (Figure 3) was introduced for the management of multiple high-powered magnetic ingestion in the pediatric population.

## Conclusion

Ingestion of multiple high-powered Buckyballs in children may lead to a high risk of severe gastrointestinal injuries, which need prompt decision-making and surgical intervention (30). In unwitnessed cases, a vague medical history and presentation of nonspecific symptoms often make the diagnosis difficult. The management options should include conservative observation, removal of the Buckyballs with primary gastrointestinal repair *via* endoscopic, laparoscopic, laparoscopy-assisted, or open surgical procedure. Minimally invasive approaches might be one of the choices (31). Taking preventive measures, such as restrictions on Buckyball manufacture and sales, health education *via* media and newspapers, and children's not having access to Buckyballs, are essential to prevent this kind of injury.

## Data Availability Statement

The raw data supporting the conclusions of this article will be made available by the authors, without undue reservation.

## Ethics Statement

Ethical review and approval was not required for the study on human participants in accordance with the local legislation and institutional requirements. Written informed consent to participate in this study was provided by the participants' legal guardian/next of kin.

## Author Contributions

GD, LG, ZB, and TF contributed to the conception and designed the study. GD, HL, PZ, QN, WW, ZF, SZ, and ZZ organized the clinical data. GD, ZB, LG, and TF wrote the manuscript. SZ, ZZ, LG, and ZB reviewed the manuscript. All the authors contributed to revising the manuscript and approved the submitted version.

## Conflict of Interest

The authors declare that the research was conducted in the absence of any commercial or financial relationships that could be construed as a potential conflict of interest.

## Publisher's Note

All claims expressed in this article are solely those of the authors and do not necessarily represent those of their affiliated organizations, or those of the publisher, the editors and the reviewers. Any product that may be evaluated in this article, or claim that may be made by its manufacturer, is not guaranteed or endorsed by the publisher.
